# Supplementary material for: YAP1 Expression in HR+HER2− Breast Cancer: 21-Gene Recurrence Score Analysis and Public Dataset Validation
Source: Cancers (Basel). 2023 Oct 18;15(20):5034. doi: 10.3390/cancers15205034 (PMC10605327; doi:10.3390/cancers15205034)
Supplement: Supplementary file 1 [file cancers-15-05034-s001.zip › cancers-2659484-supplementary.pdf]

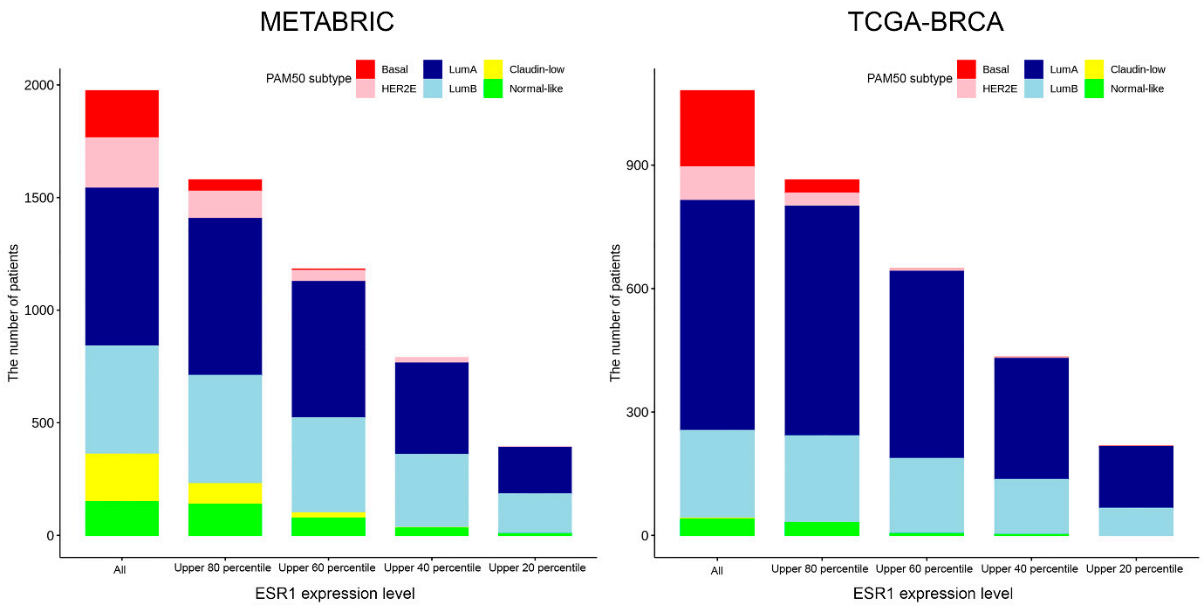

Supplementary Figure S1. Distribution of molecular subtype of breast cancer based on ESR1 expression level. LumA, luminal A; LumB, luminal B; HER2E, HER2-enriched.

Supplementary Table S1. Basal characteristics of study cohort.

Parameters	Values
Age, years (median, range)	49 (25–81)
Menopause, *n* (%)	
Premenopausal	235 (58.6)
Menopause	155 (38.7)
Not assessable	11 (2.7)
Histologic diagnosis, *n* (%)	
Invasive ductal carcinoma, NOS	365 (91.0)
Invasive lobular carcinoma	28 (7.0)
Mixed invasive ductal carcinoma	6 (1.5)
Mucinous carcinoma	2 (0.5)
Tumor size, cm (mean ± SD)	1.9 ± 0.8
Pathologic T stage	
T1	246 (61.3)
T2/3	155 (38.6)
Oncotype Dx RS (mean ± SD)	18.1 ± 9.1
Oncotype Dx risk category	
Low risk (<26)	340 (84.8)
High risk (≥26)	61 (15.2)
Histologic grade	
I	63 (15.7)
I	311 (77.6)
III	27 (6.7)
Lymphovascular invasion	
Present	122 (30.4)
Absent	279 (69.6)
Lymph node metastasis	
Present	96 (23.9)
Absent	305 (76.1)

TIL, % (mean ± SD)	11.2 ± 13.7
Two-tiered TIL group, *n* (%)	
Low-TIL (≤10%)	302 (75.3)
High-TIL (>10%)	99 (24.7)
TSR, % (mean ± SD)	71.0 ± 18.1
Two-tiered TSR group, *n* (%)	
Stroma-low (TSR >50%)	344 (86.0)
Stroma-high (TSR ≤50%)	56 (14.0)
YAP1 expression	
Low (score 0/1)	139 (34.7)
High (score 2/3)	262 (65.3)

NOS, not otherwise specified; SD, standard deviation; RS, risk score; TIL, tumor-infiltrating lymphocyte; TSR, tumor–stroma ratio; YAP1, Yes-associated protein 1.
